# The Acute and Late Toxicities of MRI-Guided External Beam Partial Breast Irradiation Delivered Using a Once-Per-Day Regimen

**DOI:** 10.3389/fonc.2021.649301

**Published:** 2021-03-23

**Authors:** Hye In Lee, Kyubo Kim, Jin Ho Kim, Ji Hyun Chang, Kyung Hwan Shin

**Affiliations:** ^1^ Department of Radiation Oncology, Seoul National University College of Medicine, Seoul, South Korea; ^2^ Department of Radiation Oncology, Ewha Womans University College of Medicine, Seoul, South Korea

**Keywords:** accelerated partial breast irradiation, once-per-day APBI, breast cancer, acute toxicity, late toxicity, MRI-guided radiotherapy

## Abstract

**Background and Purpose:**

The use of external beam accelerated partial breast irradiation (APBI) using a twice-per-day regimen has raised concerns about increase rates of late toxicities. We compared toxicity outcomes of external beam APBI using a once-per-day regimen and accelerated hypofractionated whole breast irradiation (AWBI) in patients with early-stage breast cancer.

**Materials and Methods:**

This was a single-institution, retrospective cohort study. Patients aged ≥50 years with pTisN0 or pT1N0 breast cancer who underwent breast-conserving surgery and adjuvant radiotherapy were included. APBI was delivered at 38.5 Gy in 10 fractions once daily using magnetic resonance imaging (MRI)-guided radiotherapy only to patients who were strictly “suitable”, according to the ASTRO-APBI guidelines. AWBI was delivered at 40.5–43.2 Gy in 15 or 16 fractions with or without a boost.

**Results:**

Between October 2015 and December 2018, 173 and 300 patients underwent APBI and AWBI, respectively. At a median follow-up of 34.9 months (range 7.1 to 55.4 months), the 3-year recurrence-free survival rates of the APBI and AWBI groups were both 99.2% (p=0.63). Acute toxicities were less frequent in the APBI than AWBI group (grade 1: 95 [54.9%] vs. 233 [77.7%] patients; grade 2: 7 [4.0%] vs. 44 [14.7%] patients; no grade ≥3 toxicities were observed in either group, p<0.001). Late toxicities were less common in the APBI than AWBI group (grade 1: 112 [64.7%] vs. 197 [65.7%] patients; grade 2: 9 [5.2%] vs. 64 [21.3%] patients; grade 3: 0 vs. 5 [1.7%] patients, p<0.001). Multivariate analysis showed that APBI was significantly associated with fewer late toxicities of grade ≥2 compared with AWBI (odds ratio 4.17, p=0.006).

**Conclusion:**

Once-per-day APBI afforded excellent locoregional control and fewer toxicities compared with AWBI. This scheme could be an attractive alternative to AWBI in patients who meet the ASTRO-APBI guidelines.

## Introduction

Accelerated partial breast irradiation (APBI) is a new local treatment paradigm for early-stage breast cancer. APBI is based on the finding that most cases of local recurrence develop near the original tumor bed in the treated breast (1–3). Thus, APBI-mediated delivery of radiation to the tumor bed with an adequate margin in surrounding tissue might reduce side effects while maintaining the high local control rate afforded by whole-breast irradiation. APBI spares healthy tissue, reduces treatment times, and minimizes the treatment burdens imposed on patients and healthcare systems. Since the American Society for Radiation Oncology (ASTRO) consensus statement and the Groupe European de Curietherapie–European Society for Therapeutic Radiology and Oncology recommendations proposed in 2009, APBI has been widely used in clinical practice (4, 5).

Several randomized trials and meta-analyses have reported comparable oncological outcomes when APBI is delivered *via* interstitial or balloon-based brachytherapy or intraoperative or external beam radiotherapy (6–11). External beam APBI is an attractive treatment modality that is widely available and non-invasive, and it enables accurate planning using pathological information. Two randomized trials (RAPID and NSABP B-39/RTOG 0413 trials) recently reported the outcomes of external beam APBI in more than 6,000 patients followed up for 8–10 years (12, 13). The NSABP B-39/RTOG 0413 trial reported that the local control rate and late toxicity profile of APBI were comparable with those of whole-breast irradiation. In the RAPID trial, APBI was non-inferior to whole-breast irradiation in terms of preventing ipsilateral breast tumor recurrence, but the rates of grade ≥2 late toxicities and adverse cosmesis increased with use of APBI compared with whole-breast irradiation. The investigators suggested that the increased rate of late toxicities was attributed to the twice-per-day regimen of APBI.

Our institution has performed APBI *via* magnetic resonance imaging (MRI)-guided external beam radiotherapy using a once-per-day regimen since October 2015. Here, we compared this regimen to accelerated, hypofractionated whole-breast irradiation (AWBI) as treatments for early-stage breast cancer.

## Materials and Methods

### Patient Eligibility

We retrospectively reviewed patients aged ≥50 years with pathological stage TisN0 or T1N0 breast cancer who underwent breast-conserving surgery and adjuvant radiotherapy (APBI or AWBI) between October 2015 and December 2018. Patients with other malignancies at diagnosis, bilateral breast cancer, male breast cancer, or missing follow-up data were excluded. Finally, we included 473 patients. The study was approved by the Institutional Review Board of Seoul National University Hospital (IRB no. H-2003-228-1115).

The criteria for patients “suitable” for APBI according to the ASTRO-APBI guidelines are as follows: age ≥50 years, tumor size ≤2 cm, pathological N0 status, estrogen receptor positivity, resection margin ≥2 mm, a unicentric tumor, and no lymphovascular invasion for invasive ductal carcinoma (IDC); and a tumor size ≤2.5 cm, a screen-detected tumor, a low-to-intermediate-grade tumor, and a resection margin ≥3 mm for ductal carcinoma *in situ* (DCIS) (14). We added histologic grade I/II and luminal A subtype to these inclusion criteria. However, there were a few exceptions: we included patients with mucinous carcinoma (which has a favorable prognosis) and those with smaller resection margins if they were at the superficial or deep direction of tumor. All patients who met the criteria were given a detailed explanation of the expected benefits and risks of APBI in contrast to AWBI, and only patients with consent received APBI.

### Treatment

Adjuvant radiotherapy commenced 4–6 weeks after breast-conserving surgery. APBI was performed using the ViewRay platform (MRIdian, Oakwood Village, OH, USA) equipped with three ^60^Co sources and a 0.35-T MRI device. Patients in APBI group underwent both MRI and CT (2-mm slice thickness) on the same day, as part of the simulation. Patients were scanned in the supine position using a custom vacuumlock bag for arm elevation, knee support, and a body coil on the chest for MRI acquisition. APBI was initiated one week after simulation. A cine sagittal MRI is acquired before each fraction and patients were set up to the lumpectomy cavity as visualized on MRI. Then, ViewRay acquires direct real-time visualization of the lumpectomy cavity during radiation delivery at 4 frames per second, deforms the lumpectomy contour and compares it to a predefined gating boundary that is derived from the planning image. The system automatically sends a “beam off” signal if the lumpectomy contour is outside the gating boundary. This is an additional sophisticated tool to ensure accurate treatment delivery in ViewRay. A total of 38.5 Gy in 10 fractions was prescribed, unless the resection margins were very close (<0.5 mm), in which case 40 Gy in 10 fractions was prescribed. APBI was delivered once daily for 2 weeks. The clinical target volume (CTV) was defined as unequal expansion of 10–15 mm from the tumor bed or seroma after breast-conserving surgery. Expansion in any direction was determined by the resection margin status: 10 mm expansions in directions with resection margin ≥10 mm and 15 mm expansions in directions with resection margin <10 mm. The CTV was then modified to be no closer than 3 mm to the skin surface and no deeper than the interface of the anterior chest wall and pectoralis muscle. The planning target volume (PTV) was identical to the CTV, without additional margin.

AWBI was delivered *via* three-dimensional conformal radiotherapy (3D-CRT) or intensity-modulated radiotherapy (IMRT). Patients in AWBI group underwent CT simulation (3-mm slice thickness) in the supine position using a breast board with both arms above the head and the hands holding a handlebar to reduce body rotation. Inverse-planned IMRT using sliding window technique was delivered for left-sided breast, and two opposing tangential field 3D-CRT was delivered for right-sided breast. The CTV included the whole breast but not the regional nodes. The PTV included the CTV plus a 3 mm margin. AWBI was initiated three days after simulation. Patients were treated with 40.5–43.2 Gy in 15 or 16 fractions, once daily, with or without a boost. In AWBI group, patients with invasive carcinoma or high-risk DCIS (close or positive resection margins or high-grade) were indicated for boost radiation and received boost radiation at 9–12 Gy in 3–5 fractions. The following guidelines were adopted for plan optimization in both APBI and AWBI: (1) the 95% isodose surface should cover the 100% of the PTV; (2) the maximum dose should not exceed 110% of the prescribed dose; (3) ipsilateral lung, not > 20% received a dose >20 Gy (V20 < 20%); (4) heart, mean dose <10 Gy. Patients were asked to breathe shallowly during simulation and treatment. All plans were reviewed and evaluated by a board-certified medical physicist to ascertain clinically requisite plan quality. Adjuvant chemotherapy, hormonal therapy, and anti-HER2 therapy were administered as indicated.

### Follow-Up

Patients were evaluated weekly during the course of radiotherapy and at 6 months, 1 year, and 2 years after completion of therapy. At each visit, history-taking, a physical examination, and a toxicity evaluation were performed. Mammography, breast ultrasonography, (optional) chest and breast MRI, chest and abdominal computed tomography, and a bone scan were also performed at each visit. All treatment decisions, planning, follow-up, and toxicity evaluations were performed by a single radiation oncologist (KHS) over the entire 3-year study period.

### Outcomes

Ipsilateral breast tumor recurrence (IBTR) was defined as histological evidence of invasive or *in situ* disease in the ipsilateral breast. IBTR was described as true recurrence if it developed within 2 cm of the tumor bed or as elsewhere recurrence otherwise. Recurrence-free survival was the time from commencement of radiotherapy to any documented recurrence in the ipsilateral breast, regional lymph node, or a distant site. Event-free survival was defined as the time from commencement of radiotherapy to any documented recurrence, contralateral breast cancer, or death. Acute toxicity was defined as toxicity observed during the radiotherapy period and late toxicity as toxicity observed during routine follow-up no earlier than 6 months after radiotherapy. Acute and late toxicities were graded using the Common Terminology Criteria for Adverse Events (CTCAE), ver. 4.0.

### Statistical Analysis

The recurrence-free and event-free survival rates were estimated using the Kaplan–Meier method. Univariate and multivariate logistic regression analyses were employed to seek associations between various factors and late toxicities. Statistical significance was defined by a p-value <0.05. All statistical analyses were performed using STATA software ver. 16.0 (StataCorp LP, College Station, TX, USA).

## Results

### Patient Characteristics

Between October 2015 and December 2018, 473 patients were included, of whom 173 and 300 received APBI and AWBI, respectively. **Table 1** lists the patient, tumor, and treatment-related characteristics of both groups. The median age was 61 years (range 51–81 years) in the APBI group and 58 years (range 50–80 years) in the AWBI group. In the APBI group, 166 (96%) patients had IDC, 5 (3%) DCIS, and 2 (1%) mucinous carcinoma. In the AWBI group, 226 (75%) had IDC, 54 (18%) DCIS, and 20 (7%) other pathologies. The median tumor size was 1.2 cm (range 0.1–2.0 cm) in the APBI group and 1.1 cm (range 0.1–2.0 cm) in the AWBI group. In the APBI group, all patients were estrogen receptor-positive and received endocrine therapy. None received chemotherapy or anti-HER2 therapy. APBI was performed using the ViewRay platform (MRI-guided radiotherapy); 38.5 Gy was delivered in 10 fractions without any boost. Two (0.7%) patients received 40 Gy in 10 fractions because they had very close superficial resection margins (<0.5 mm). In the AWBI group, 204 (68%) patients were estrogen receptor-positive, and 206 (69%) patients received endocrine therapy, 91 (30%) chemotherapy, and 34 (11%) anti-HER2 therapy. Whole-breast irradiation was delivered *via* 3D-CRT to 150 (50%) patients and *via* IMRT to 150 (50%) patients [43.2 Gy in 16 fractions to 195 (65%) and 40.5 Gy in 15 fractions to 105 (35%)]. A total of 257 (86%) patients received boosts (9–12 Gy in 3–5 fractions). The median follow-up time was 34.9 months (range 7.1–55.4 months).

**Table 1:** Patient, treatment, and tumor-related characteristics of all patients.

Characteristics		APBI (N=173)		AWBI (N=300)		p-value
Age at diagnosis (years)	Median (range)	61	(51–81)	58	(50–80)	
Histology						<0.001
	DCIS	5	(3%)	54	(18%)	
	IDC	166	(96%)	226	(75%)	
	ILC	0	(0%)	17	(6%)	
	Other	2	(1%)	3	(1%)	
Tumor size						0.881
	<1.5 cm	102	(59%)	179	(60%)	
	≥1.5 cm	71	(41%)	121	(40%)	
Histologic grade						<0.001
	I (low)	44	(25%)	38	(13%)	
	II (intermediate)	125	(72%)	161	(54%)	
	III (high)	4	(2%)	101	(33%)	
Estrogen receptor status						<0.001
	Positive	173	(100%)	204	(68%)	
	Negative	0	(0%)	96	(32%)	
HER2 status						<0.001
	Positive	1	(1%)	72	(24%)	
	Negative	172	(99%)	228	(76%)	
Resection margin						<0.001
	Negative	147	(85%)	198	(66%)	
	Close	26	(15%)	87	(29%)	
	Positive	0	(0%)	15	(5%)	
Chemotherapy						<0.001
	Yes	0	(0%)	91	(30%)	
	No	173	(100%)	209	(70%)	
Endocrine therapy						<0.001
	Tamoxifen	56	(32%)	98	(33%)	
	Aromatase inhibitor	117	(68%)	108	(36%)	
	None	0	(0%)	94	(31%)	
Anti-HER2 therapy						<0.001
	Yes	0	(0%)	34	(11%)	
	No	173	(100%)	266	(89%)	
Boost radiation						<0.001
	Yes	0	(0%)	257	(86%)	
	No	173	(100%)	43	(14%)	

DCIS, ductal carcinoma in situ; IDC, invasive ductal carcinoma; ILC, invasive lobular carcinoma; HER2, human epidermal growth factor receptor 2; APBI, accelerated partial breast irradiation; AWBI, accelerated whole-breast irradiation.

### Recurrence and Survival

**Figure 1:**
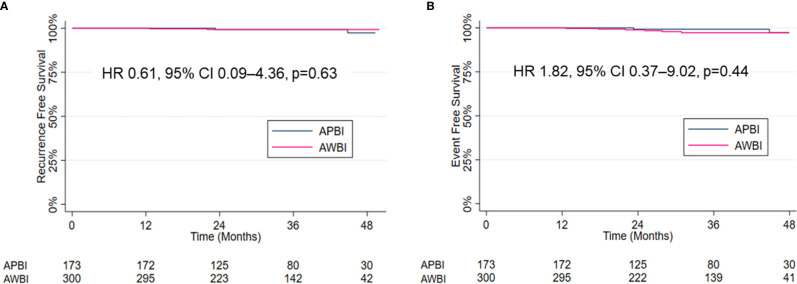
Kaplan–Meier estimates and curves for recurrence-free survival **(A)** and event-free survival **(B)**.

Four patients developed recurrence. In the APBI group, two (1.2%) patients experienced IBTRs and both were elsewhere recurrences. The disease-free intervals from the commencement of radiotherapy were 23 and 45 months, respectively. In the AWBI group, one (0.3%) patient developed regional recurrence in the ipsilateral supraclavicular lymph node and one (0.3%) distant recurrence in multiple bones and the lung. The disease-free intervals were 13 and 22 months, respectively. Four (1.3%) patients in the AWBI group developed contralateral breast cancer at 18, 25, 28, and 31 months after commencement of radiotherapy, respectively, and one of them died of an unknown cause. The 3-year recurrence-free survival rates in the APBI and AWBI groups were both 99.2% (**Figure 1A**, hazard ratio [HR] 0.61, 95% confidence interval [CI] 0.09–4.36, p=0.63). The 3-year event-free survival rates of the APBI and AWBI groups were 99.2% and 97.2% respectively (**Figure 1B**, HR 1.82, 95% CI 0.37–9.02, p=0.44). The event types by treatment group are shown in **Table 2**, and detailed information is given in **Table 3**. There was no significant difference between the two groups in recurrence-free survival and event-free survival.

**Table 2:** Event type by treatment group.

	APBI (N=173)		AWBI (N=300)	
Ipsilateral breast tumor recurrence	2	(1.15%)	0	(0.00%)
Regional recurrence	0	(0.00%)	1	(0.33%)
Distant recurrence	0	(0.00%)	1	(0.33%)
Contralateral breast cancer	0	(0.00%)	4	(1.33%)
Death	0	(0.00%)	1	(0.33%)
Recurrence total	2	(1.15%)	2	(0.66%)
Event total	2	(1.15%)	6	(2.00%)

APBI, accelerated partial breast irradiation; AWBI, accelerated whole-breast irradiation.

**Table 3:** Detailed information on patients who experienced any event.

No	Age	Primary site	Stage	Molecular subtype	Histologic grade	Resection margin	Radiotherapy	Other treatment	Failure pattern	Recurrence site	Disease-free interval (months)	Survival
1	63	Lt breast	pT1cN0	Luminal A	III	1.2 cm (deep)	APBI	Tamoxifen	IBTR, elsewhere	Elsewhere, Lt breast	23.3	Alive
2	57	Lt breast	pT1bN0	Luminal A	II	0.2 cm (deep)	APBI	Tamoxifen	IBTR, elsewhere	Elsewhere, Lt breast	44.8	Alive
3	57	Rt breast	pT1bN0	TNBC	III	<0.1 cm (superf)	AWBI	Neoadj chemo	Contralateral BC	Lt breast	25.1	Alive
4	63	Lt breast	pT1cN0	Luminal A	III	0.8 cm (deep)	AWBI	Aromatase inhibitor	Distant recurrence	Bones & lung	22.0	Alive
5	51	Lt breast	pT1aN0	Luminal A	II	0.2 cm (deep)	AWBI	Tamoxifen	Contralateral BC	Rt breast	30.9	Alive
6	57	Lt breast	pT1aN0	HER2-enriched	III	0.5 cm (lateral)	AWBI	–	Contralateral BC	Rt breast	17.8	Alive
7	50	Rt breast	pT1bN0	Luminal A	II	0.8 cm (deep)	AWBI	Tamoxifen	Contralateral BC	Lt breast	27.9	Dead
8	63	Lt breast	pT1cN0	HER2-enriched	II	<0.1 cm (inferior)	AWBI	Adj chemo + anti-HER2 therapy	Regional recurrence	Lt SCN	12.6	Alive

TNBC, triple-negative breast cancer; HER2, Human epidermal growth factor receptor 2; APBI, accelerated partial breast irradiation; AWBI, accelerated whole-breast irradiation; Neoadj chemo, neoadjuvant chemotherapy; Adj chemo, adjuvant chemotherapy; IBTR, ipsilateral breast tumor recurrence; BC, breast cancer; SCN, supraclavicular lymph node.

### Acute and Late Toxicities

**Figure 2:**
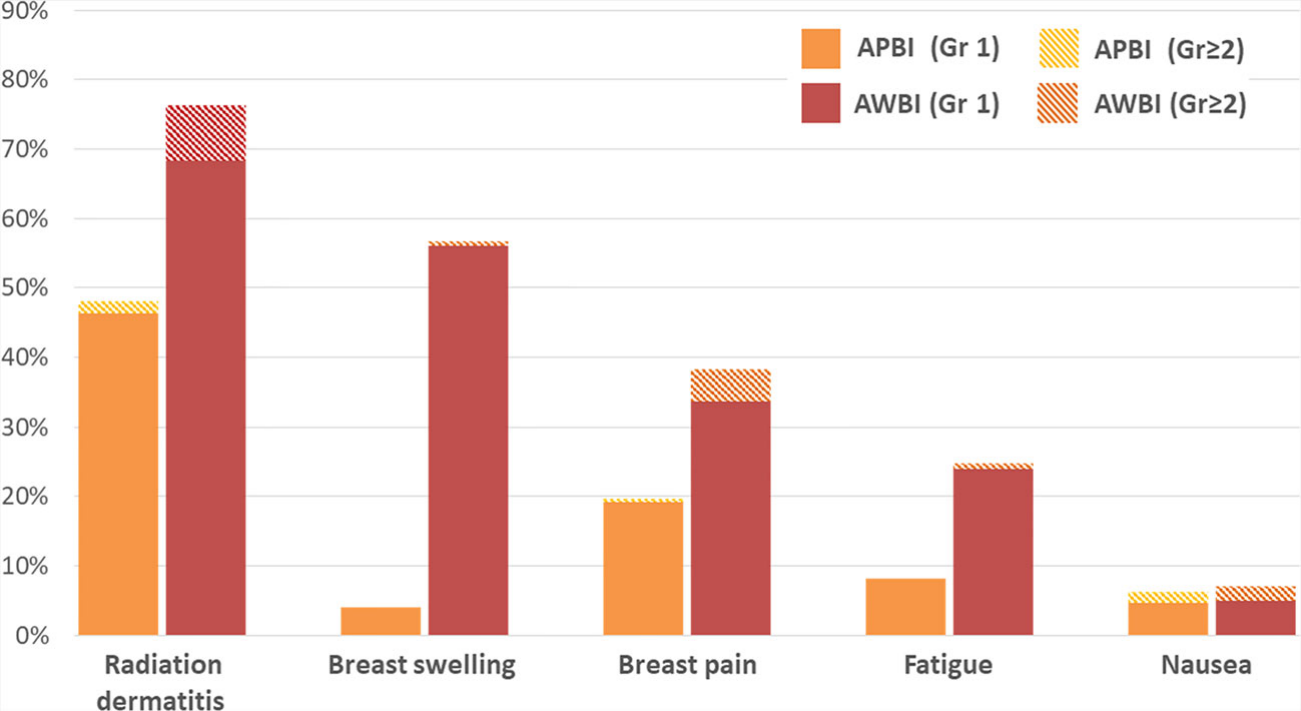
Acute toxicities (during the radiotherapy period) by treatment group.

The acute and late radiation toxicities are presented in **Table 4**. Acute toxicities were less common in the APBI than AWBI group (grade 1: 95 [54.9%] vs. 233 [77.7%] patients; grade 2: 7 [4.0%] vs. 44 [14.7%] patients; no grade ≥3 toxicities in either group). During the radiotherapy period, radiation dermatitis was the most common toxicity, followed by breast swelling and breast pain. Radiation dermatitis occurred in 83 (48%) patients in the APBI group and 229 (76.3%) in the AWBI group. Of these patients, 3 (1.7%) in the APBI group and 24 (8.0%) in the AWBI group developed grade 2 dermatitis and were managed with antibiotics or steroid creams. Seven (4.0%) patients in the APBI group and 168 (56.0%) in the AWBI group experienced breast swelling, all of grade 1. Thirty-four (19.7%) patients in the APBI group and 114 (38.0%) in the AWBI group experienced breast pain, of whom 1 (0.6%) in the APBI group and 13 (4.3%) in the AWBI group took oral analgesics. Additionally, three (1.7%) patients in the APBI group and six (2.0%) in the AWBI group took anti-emetics because of nausea during radiotherapy. No toxicity of grade ≥3 developed in either group. **Figure 2** shows the acute toxicities in both treatment groups; APBI was associated with lower rates of all acute toxicity categories compared with AWBI.

**Table 4:** Acute and late radiation toxicities by treatment group.

	APBI (N = 173)								AWBI (N = 300)							
	Grade 1		Grade 2		Grade 3		Total		Grade 1		Grade 2		Grade 3		Total	
Acute period																
Dermatitis	80	(46.2%)	3	(1.7%)	0	(0.0%)	83	(48.0%)	205	(68.3%)	24	(8.0%)	0	(0.0%)	229	(76.3%)
Breast swelling	7	(4.0%)	0	(0.0%)	0	(0.0%)	7	(4.0%)	168	(56.0%)	0	(0.0%)	0	(0.0%)	168	(56.0%)
Breast pain	33	(19.1%)	1	(0.6%)	0	(0.0%)	34	(19.7%)	101	(33.7%)	13	(4.3%)	0	(0.0%)	114	(38.0%)
Fatigue	14	(8.1%)	0	(0.0%)	0	(0.0%)	14	(8.1%)	72	(24.0%)	2	(0.7%)	0	(0.0%)	74	(24.7%)
Nausea	8	(4.6%)	3	(1.7%)	0	(0.0%)	11	(6.4%)	15	(5.0%)	6	(2.0%)	0	(0.0%)	21	(7.0%)
Late period																
Breast pain	74	(42.8%)	3	(1.7%)	0	(0.0%)	77	(44.5%)	110	(36.7%)	21	(7.0%)	0	(0.0%)	131	(43.7%)
Pigmentation	43	(24.9%)	0	(0.0%)	0	(0.0%)	43	(24.9%)	142	(47.3%)	2	(0.7%)	0	(0.0%)	144	(48.0%)
Breast swelling	9	(5.2%)	0	(0.0%)	0	(0.0%)	9	(5.2%)	117	(39.0%)	8	(2.7%)	0	(0.0%)	125	(41.7%)
Dermatitis	30	(17.3%)	5	(2.9%)	0	(0.0%)	35	(20.2%)	30	(10.0%)	19	(6.3%)	2	(0.7%)	51	(17.0%)
Fibrosis	16	(9.2%)	0	(0.0%)	0	(0.0%)	16	(9.2%)	35	(11.7%)	2	(0.7%)	0	(0.0%)	37	(12.3%)
Rib change	9	(5.2%)	0	(0.0%)	0	(0.0%)	9	(5.2%)	14	(4.7%)	0	(0.0%)	0	(0.0%)	14	(4.7%)
Telangiectasia	5	(2.9%)	1	(0.6%)	0	(0.0%)	6	(3.5%)	13	(4.3%)	0	(0.0%)	0	(0.0%)	13	(4.3%)
Lymphedema	4	(2.3%)	0	(0.0%)	0	(0.0%)	4	(2.3%)	10	(3.3%)	0	(0.0%)	0	(0.0%)	10	(3.3%)
Pneumonitis	0	(0.0%)	2	(1.2%)	0	(0.0%)	2	(1.2%)	0	(0.0%)	6	(2.0%)	3	(1.0%)	9	(3.0%)
Fatty necrosis	4	(2.3%)	0	(0.0%)	0	(0.0%)	4	(2.3%)	4	(1.3%)	0	(0.0%)	0	(0.0%)	4	(1.3%)

APBI, accelerated partial breast irradiation; AWBI, accelerated whole-breast irradiation.

**Figure 3:**
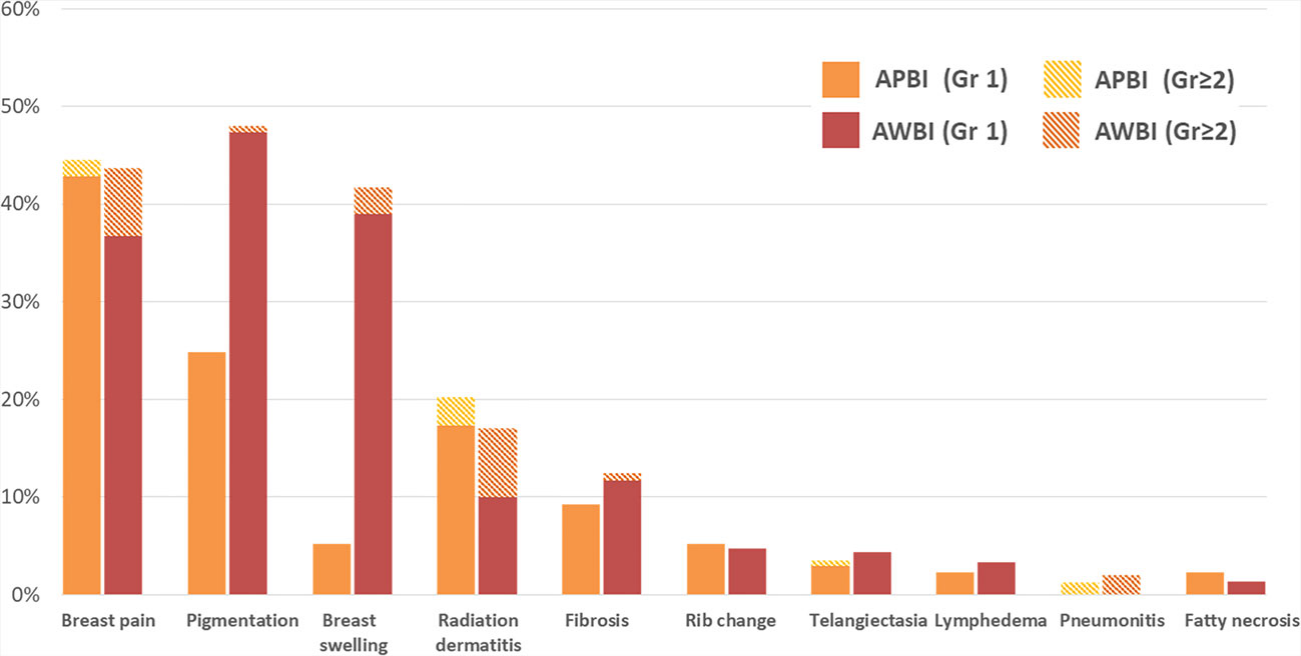
Late toxicities (at least 6 months after radiotherapy) by treatment group.

In terms of late toxicities (those developing at least 6 months after radiotherapy commencement), the rates in the APBI group were similar to or lower than those in the AWBI group (grade 1: 112 [64.7%] vs. 197 [65.7%] patients; grade 2: 9 [5.2%] vs. 64 [21.3%] patients; grade 3: 0 vs. 5 [1.7%] patients). Breast pain, pigmentation, and swelling were observed in >20% of all patients. Breast pain, which was the most common late toxicity, was observed in 77 (44.5%) patients in the APBI group and 131 (43.7%) in the AWBI group. Of these patients, 3 (1.7%) in the APBI group and 21 (7.0%) in the AWBI group required analgesics. Forty-three (24.9%) patients in the APBI group and 142 (47.3%) in the AWBI developed mild pigmentation, and 2 (0.7%) in the AWBI group developed moderate pigmentation. Nine (5.2%) patients in the APBI group developed grade 1 breast swelling, and 117 (39.0%) and 8 (2.7%) in the AWBI group developed breast swelling of grades 1 and 2, respectively. Symptomatic rib changes evident on bone scans were reported in 9 (5.2%) patients in the APBI group and 14 (4.7%) in the AWBI group. No grade 3 late toxicities were observed in the APBI group, but five (1.7%) patients in the AWBI group developed such grade 3 radiation pneumonitis (n=3) and wound complications (n=2). The patients with radiation pneumonitis were hospitalized and given intravenous antibiotics. One patient with a grade 3 wound complication was diagnosed with breast cellulitis and bacteremia and was given intravenous antibiotics during repeat hospitalization. Another patient with grade 3 wound complications underwent re-operation because of breast cellulitis. All grade 3 late toxicities resolved completely after treatment. No grade 4 or 5 late toxicities developed in either group. **Figure 3** shows the late toxicities in each treatment group.

### Analysis of Late Toxicities

The results of univariate and multivariate logistic regression analyses of factors that might contribute to late toxicities are shown in **Table S1** and **S2**. The radiotherapy technique (APBI or AWBI), boost radiation, endocrine therapy, chemotherapy, and anti-HER2 therapy were associated with late toxicities of all grades on univariate analysis (all p<0.05). Of these, only boost radiation was significantly associated with more late toxicities on multivariate analysis. For grade ≥2 late toxicities, the radiotherapy technique and boost radiation exhibited significant associations on univariate analysis, but only the radiotherapy technique was significant on multivariate analysis. The APBI group exhibited significantly fewer grade ≥2 late toxicities compared with the AWBI group, regardless of the boost radiation status (APBI vs. AWBI without boost radiation: OR 4.17, 95% CI 1.51–11.55, p=0.006; APBI vs. AWBI with boost radiation: OR 5.08, 95% CI 2.44–10.57, p<0.001). For all grades of late toxicities, the APBI group exhibited significantly fewer toxicities than those in the AWBI with boost radiation (OR 4.17, 95% CI 2.45–7.10, p<0.001), but there was no significant difference between the APBI and AWBI groups without boost radiation (OR 1.42, 95% CI 0.65–3.09, p=0.379).

### Subgroup Analysis of Acute and Late Toxicities

We conducted a subgroup analysis of 382 patients (173 in APBI group, 209 in AWBI group), excluding those who underwent chemotherapy or anti-HER2 therapy, because they might potentially bias toxicity analysis. As a result, the subgroup demonstrated almost similar results in acute toxicities (grade 1: 95 [54.9%] vs. 159 [76.1%] patients; grade 2: 7 [4.0%] vs. 32 [15.3%] patients; no grade ≥3 toxicities in either group) and late toxicities (grade 1: 112 [64.7%] vs. 135 [64.6%] patients; grade 2: 9 [5.2%] vs. 44 [21.1%] patients; grade 3: 0 vs. 3 [1.4%] patients). Detailed information on acute and late toxicities in both groups is given in **Table S3**. In the multivariate logistic regression analysis, only radiation technique (APBI or AWBI) was significantly associated with grade ≥2 late toxicities (OR 3.55, 95% CI 1.24-10.22, p=0.019) and none had significant association with all grades of late toxicities (**Tables S4**, **S5**). Therefore, APBI group exhibited significantly fewer grade ≥2 late toxicities compared with AWBI group, in both entire cohort and subgroup analysis.

## Discussion

We found that external beam APBI using a once-per-day regimen for patients who were “suitable” for APBI according to the ASTRO-APBI guidelines afforded excellent locoregional control and fewer acute and late toxicities compared with AWBI. At a median follow-up of 34.9 months, the IBTR rate was 1.2%, and no regional or distant recurrence was noted, in the APBI group. This suggests that APBI did not increase the regional or distant recurrence rate. One (0.3%) regional recurrence, one (0.3%) distant recurrence, and four (1.3%) contralateral breast cancer cases developed in the AWBI group. As that group included more unfavorable characteristics (histologic grade III, estrogen receptor-negative and/or HER2-positive tumors, or positive resection margins), we predicted that the overall prognosis would be poorer in the AWBI group than APBI group (15). However, both groups exhibited excellent outcomes, with 3-year recurrence rates <1%.

As higher skin doses have been expected for external beam APBI, concerns have been raised about skin toxicity. However, our toxicity outcomes were promising. Acute toxicity was less frequent in the APBI than AWBI group. Acute toxicity is more dependent on the total dose than the fraction size, consistent with our results (16). Although 7 (4.0%) patients in the APBI group and 44 (14.7%) in the AWBI group experienced grade 2 acute toxicities, all were successfully managed with oral or topical medications. In terms of late toxicities, the APBI group exhibited similar or lower toxicities in every category compared with the AWBI group. These results were similar when compared except for patients receiving chemotherapy or anti-HER2 therapy in AWBI group. Especially, breast swelling significantly contributed to the difference in late toxicities between the two groups (breast swelling: 9 [5.2%] patients in the APBI vs. 125 [41.7%] in the AWBI group). Young-Afat et al. reported that locoregional radiotherapy increased the risk of breast swelling, associated with breast pain and reduced quality of life (17). Generally, breast swelling per se does not require management, but interventions may be required if pain develops.

Recently, three randomized trials of external beam APBI have been reported (12, 13, 18). The RAPID trial, which randomly assigned 2,135 patients to receive either external beam APBI (38.5 Gy in 10 fractions twice daily) or whole-breast irradiation, reported more late toxicities in the APBI than whole-breast irradiation group (grade ≥2 late toxicities: 32% vs. 13%) (12). In the IRMA trial, 3,309 patients were randomly assigned to the APBI (38.5 Gy in 10 fractions twice daily) or whole-breast irradiation group. The interim results showed increased rates of late subcutaneous tissue and bone toxicities in the APBI than whole-breast irradiation group (grade 2–4 subcutaneous tissue toxicities: 44% vs. 29%; grade 3–4 bone toxicities: 1% vs. 0%) (18). The NSABP B-39/RTOG 0413 trial randomly assigned 4,216 patients to either an APBI twice-per-day regimen or whole-breast irradiation. Although the grade 2 toxicity level was slightly decreased, the grade 3 toxicity level was higher in the APBI than the whole-breast irradiation group (grade 1: 40% vs. 31%; grade 2: 44% vs. 59%; grade ≥3: 10% vs. 7%) (13). The authors concluded that the toxicity levels were similar between the two groups. However, APBI was delivered *via* brachytherapy in 27% of patients in the APBI group, which would have lowered the toxicity rate.

Our study has several different points from these three randomized trials. First, all patients in our AWBI group treated with accelerated, hypofractionated whole-breast irradiation at 40.5 Gy in 15 fractions or 43.2 Gy in 16 fractions. Most APBI trials, including the RAPID, IRMA and NSABP B-39/RTOG 0413 trials, delivered conventionally fractionated whole-breast irradiation as 50 Gy in 25 fractions to all or a subset of patients. Since the hypofractionated whole-breast irradiation represents a new standard regimen, our favorable results will be a great help in clinical practice (16). Second, unlike the three randomized trials using a twice-per-day regimen for APBI, we performed APBI using a once-per-day regimen. Several studies reported that a 6-h interval between external beam fractions was inadequate for repair of radiation injuries to healthy tissues and recommended an inter-fraction interval of ≥24 h (19–21). Yarnold et al. reported that 38.5 Gy delivered in 10 fractions using a twice-per-day regimen was equivalent to a theoretical dose of 65 Gy (2 Gy/fraction), whereas 38.5 Gy in 10 fractions delivered *via* a once-per-day regimen with α/β=3.4 Gy was equivalent to 52 Gy (2 Gy/fraction) (19). Regardless of the schedule, a twice-per-day regimen would be associated with significant biological effects caused by incomplete recovery. Meattini et al. performed APBI at 30 Gy in 5 nonconsecutive once-daily fractions, at 48-h intervals, and reported favorable toxicity outcomes of APBI (22). We performed APBI using a once-per-day regimen, thus at 24-h intervals. The late toxicity rate was lower in the APBI than AWBI group. Our result is consistent with the conclusions of the cited studies that the inter-fraction interval greatly influences the outcomes of late toxicities.

Boost radiation is known to adversely affect toxicities and the cosmetic results (23, 24). Coles et al. compared the RAPID and NSABP B-39/RTOG 0413 trials and suggested that the use of boost radiation in the whole-breast irradiation group might have contributed to the observed differences in toxicity (25). In the NSABP B-39/RTOG 0413 trial, 80% of patients in the whole-breast irradiation group received boost radiation, compared with only 21% in the RAPID trial. In our study, 257 (86%) patients in AWBI group received boost radiation. Using an α/β ratio of 3.5 Gy for late toxicity, the equivalent doses in 2 Gy fraction (EQD2) of AWBI, boost radiation and APBI are 45.7-48.7 Gy, 10.6-12.9 Gy, and 51.5 Gy, respectively. Using an α/β ratio of 10 Gy for acute toxicity, the EQD2 of AWBI, boost radiation and APBI are 42.9-45.7 Gy, 9.8-12.4 Gy and 44.4 Gy, respectively. Thus, the EQD2 of AWBI without boost radiation is similar or lower than that of APBI, but for AWBI with boost radiation, the EQD2 is clearly higher than that of APBI. This is consistent with our toxicity results. AWBI patients who did not receive boost radiation did not differ from the APBI group in terms of late toxicity of all grades and only AWBI patients who receive boost radiation had significantly higher rates of late toxicity of all grades. Although the rate of late toxicities of grade ≥2 was higher in the AWBI group regardless of the boost radiation status, it is clear that boost radiation contributed to some extent to the between-group difference in late toxicities.

To our knowledge, this study is the largest trial that performed APBI using the MRIdian, ViewRay. ViewRay ensures more accurate treatment delivery by improved visualization with superior soft tissue contrast of MRI, daily set up to the lumpectomy cavity as visualized on a cine MRI, and a real-time motion tracking and gating system. This makes it possible to reduce the PTV margin (26–29). Acharya et al. analyzed interfractional motion of the breast lumpectomy cavity in ViewRay, and reported that a mean PTV margin of 0.7 mm would be sufficient to cover 90% of the lumpectomy cavity for 90% of the treatment time (30). In this study, despite using no PTV margins, the mean difference in dose planned vs. delivered was only 1%, further supporting reduced PTV margins with ViewRay. As we applied different CTV margins (10-15 mm) according to the directional safety margin status, we regarded that the differential CTV margin obviate the need of PTV margin. For these reasons, we applied no additional PTV margin from the CTV and reduced irradiated volume, while maintaining the high local control rate. Since only few outcomes of APBI using MRI-guided radiotherapy have been published, our findings add valuable knowledge for this.

On the other hand, our work had certain limitations. This was a retrospective single-institutional study with a relatively short follow-up period. The group characteristics differed because we applied the strict ASTRO-APBI guidelines to the APBI group. Chemotherapy and anti-HER2 therapy were prescribed only for indicated patients in the AWBI group; these might have increased the acute and late toxicities. However, in our subgroup analysis excluding those who underwent chemotherapy or anti-HER2 therapy, the acute and late toxicities were almost similar with the entire cohort, and APBI group still demonstrated significantly fewer late toxicities compared with AWBI group. Finally, we did not assess cosmetic outcomes; however, most patients were satisfied with their cosmesis.

Currently, the results of other randomized trials of external beam APBI are pending; however, most performed APBI twice per day (31, 32). We show that it is safe to use APBI once per day, although a prospective randomized trial is required. As a thorough assessment of late toxicities after APBI requires at least 5 years of follow-up, we will report the results of longer-term follow-up later, and we hope that these will aid the establishment of an optimal APBI scheme.

## Conclusion

The results of our single-institution retrospective study showed that external beam APBI delivered once-per-day is an effective option for patients with early-stage breast cancer. The recurrence rate is extremely low, and the incidences of acute and late toxicities are both low. This scheme can be safely recommended for early-stage breast cancer patients who meet the ASTRO-APBI guidelines.

## Data Availability Statement

The raw data supporting the conclusions of this article will be made available by the authors, without undue reservation.

## Author Contributions

KS contributed conception and design of the study. HL contributed to data collection, statistical analysis and wrote the first draft of the manuscript. KK and KS revised the manuscript. All authors contributed to the article and approved the submitted version.

## Conflict of Interest

The authors declare that the research was conducted in the absence of any commercial or financial relationships that could be construed as a potential conflict of interest.
