# Supplementary material for: The Acute and Late Toxicities of MRI-Guided External Beam Partial Breast Irradiation Delivered Using a Once-Per-Day Regimen
Source: Front Oncol. 2021 Mar 23;11:649301. doi: 10.3389/fonc.2021.649301 (PMC8021959; doi:10.3389/fonc.2021.649301)
Supplement: Supplementary file 1 [file Table_1.docx]

Table S1: Prognostic factors for late toxicity.

|  | |  |  | Univariate analysis | | | Multivariate analysis | | |
| --- | --- | --- | --- | --- | --- | --- | --- | --- | --- |
|  |  | No. of events | No. of patients | OR | 95% CI | p-value | OR | 95% CI | p-value |
| Age |  |  |  |  |  | 0.738 |  |  |  |
|  | <60 years | 277 | 337 | 1 | Reference |  |  |  |  |
|  | ≥60 years | 110 | 136 | 0.92 | 0.55-1.53 |  |  |  |  |
| Histology |  |  |  |  |  | 0.446 |  |  |  |
|  | DCIS | 51 | 59 | 1 | Reference |  |  |  |  |
|  | IDC | 316 | 392 | 0.66 | 0.30-1.45 |  |  |  |  |
|  | Other | 20 | 22 | 1.05 | 0.25-4.34 |  |  |  |  |
| Tumor size |  |  |  |  |  | 0.613 |  |  |  |
|  | <1.5 cm | 232 | 281 | 1 | Reference |  |  |  |  |
|  | ≥1.5 cm | 155 | 192 | 0.88 | 0.55-1.42 |  |  |  |  |
| Axillary LN assessment |  |  |  |  |  | 0.657 |  |  |  |
|  | None | 30 | 38 | 1 | Reference |  |  |  |  |
|  | SLNB | 353 | 431 | 1.2 | 0.53-2.73 |  |  |  |  |
|  | ALND | 4 | 4 | - | - |  |  |  |  |
| Radiotherapy technique |  |  |  |  |  | <0.001 |  |  | 0.409 |
|  | APBI | 121 | 173 | 1 | Reference |  | 1 | Reference |  |
|  | AWBI | 266 | 300 | 3.36 | 2.07-5.45 |  | 1.39 | 0.64–3.04 |  |
| Boost radiation |  |  |  |  |  | <0.001 |  |  | 0.050 |
|  | No | 154 | 216 | 1 | Reference |  | 1 | Reference |  |
|  | Yes | 233 | 257 | 3.91 | 2.34-6.53 |  | 2.36 | 1.00–5.59 |  |
| Endocrine therapy |  |  |  |  |  | 0.003 |  |  | 0.921 |
|  | No | 86 | 94 | 1 | Reference |  | 1 | Reference |  |
|  | Yes | 301 | 379 | 0.36 | 0.16-0.77 |  | 0.95 | 0.39–2.33 |  |
| Chemotherapy |  |  |  |  |  | <0.001 |  |  | 0.194 |
|  | No | 301 | 382 | 1 | Reference |  | 1 | Reference |  |
|  | Yes | 86 | 91 | 4.63 | 1.82-11.78 |  | 2.34 | 0.65–8.47 |  |
| Anti-HER2 therapy |  |  |  |  |  | 0.030 |  |  | 0.884 |
|  | No | 355 | 439 | 1 | Reference |  | 1 | Reference |  |
|  | Yes | 32 | 34 | 3.79 | 0.89-16.11 |  | 0.87 | 0.14–5.51 |  |

Abbreviations: DCIS, ductal carcinoma in situ; IDC, invasive ductal carcinoma; LN, lymph node; SLNB, sentinel lymph node biopsy; ALND, axillary lymph node dissection; APBI, accelerated partial breast irradiation; AWBI, accelerated whole-breast irradiation; HER2, human epidermal growth factor receptor 2.

Table S2: Prognostic factors for grade ≥2 late toxicity.

|  | |  |  | Univariate analysis | | | Multivariate analysis | | |
| --- | --- | --- | --- | --- | --- | --- | --- | --- | --- |
|  |  | No. of events | No. of patients | OR | 95% CI | p-value | OR | 95% CI | p-value |
| Age |  |  |  |  |  | 0.573 |  |  |  |
|  | <60 years | 54 | 337 | 1 | Reference |  |  |  |  |
|  | ≥60 years | 19 | 136 | 0.85 | 0.48–1.49 |  |  |  |  |
| Histology |  |  |  |  |  | 0.143 |  |  |  |
|  | DCIS | 12 | 59 | 1 | Reference |  |  |  |  |
|  | IDC | 60 | 391 | 0.71 | 0.35–1.42 |  |  |  |  |
|  | Other | 1 | 23 | 0.18 | 0.02–1.45 |  |  |  |  |
| Tumor size |  |  |  |  |  | 0.541 |  |  |  |
|  | <1.5 cm | 41 | 281 | 1 | Reference |  |  |  |  |
|  | ≥1.5 cm | 32 | 192 | 1.17 | 0.71–1.94 |  |  |  |  |
| Axillary LN assessment |  |  |  |  |  | 0.549 |  |  |  |
|  | None | 8 | 38 | 1 | Reference |  |  |  |  |
|  | SLNB | 64 | 431 | 0.65 | 0.29–1.49 |  |  |  |  |
|  | ALND | 1 | 4 | 1.25 | 0.11–13.69 |  |  |  |  |
| Radiotherapy technique |  |  |  |  |  | <0.001 |  |  | 0.006 |
|  | APBI | 9 | 173 | 1 | Reference |  | 1 | Reference |  |
|  | AWBI | 64 | 300 | 4.94 | 2.39–10.21 |  | 4.17 | 1.50–11.55 |  |
| Boost radiation |  |  |  |  |  | <0.001 |  |  | 0.637 |
|  | No | 17 | 216 | 1 | Reference |  | 1 | Reference |  |
|  | Yes | 56 | 257 | 3.26 | 1.83–5.81 |  | 1.22 | 0.54–2.78 |  |
| Endocrine therapy |  |  |  |  |  | 0.094 |  |  |  |
|  | No | 21 | 94 | 1 | Reference |  |  |  |  |
|  | Yes | 52 | 379 | 0.55 | 0.31–0.97 |  |  |  |  |
| Chemotherapy |  |  |  |  |  | 0.064 |  |  |  |
|  | No | 53 | 382 | 1 | Reference |  |  |  |  |
|  | Yes | 20 | 91 | 1.75 | 0.98–3.11 |  |  |  |  |
| Anti-HER2 therapy |  |  |  |  |  | 0.902 |  |  |  |
|  | No | 68 | 439 | 1 | Reference |  |  |  |  |
|  | Yes | 5 | 34 | 0.94 | 0.35–2.52 |  |  |  |  |

Abbreviation: DCIS, ductal carcinoma in situ; IDC, invasive ductal carcinoma; LN, lymph node; SLNB, sentinel lymph node biopsy; ALND, axillary lymph node dissection; APBI, accelerated partial breast irradiation; AWBI, accelerated whole-breast irradiation; HER2, human epidermal growth factor receptor 2.

Table S3: Acute and late radiation toxicities in subgroup analysis (excluding patients who underwent chemotherapy or anti-HER2 therapy)

|  | APBI (N=173) | | | | | | | | AWBI (N=209) | | | | | | | | | | | |
| --- | --- | --- | --- | --- | --- | --- | --- | --- | --- | --- | --- | --- | --- | --- | --- | --- | --- | --- | --- | --- |
|  | Grade 1 | | Grade 2 | | Grade 3 | | Total | | Grade 1 | | Grade 2 | | | Grade 3 | | | | Total | | |
| Acute period | | | | | | | | | | | | | | | | | | | | |
| Dermatitis | 80 | (46.2%) | 3 | (1.7%) | 0 | (0.0%) | 83 | (48.0%) | 140 | (67.0%) | 13 | (6.2%) | | 0 | (0.0%) | | 153 | | (73.2%) | |
| Breast swelling | 7 | (4.0%) | 0 | (0.0%) | 0 | (0.0%) | 7 | (4.0%) | 117 | (56.0%) | 0 | (0.0%) | | 0 | (0.0%) | | 117 | | (56.0%) | |
| Breast pain | 33 | (19.1%) | 1 | (0.6%) | 0 | (0.0%) | 34 | (19.7%) | 69 | (33.0%) | 12 | (5.7%) | | 0 | (0.0%) | | 81 | | (38.8%) | |
| Fatigue | 14 | (8.1%) | 0 | (0.0%) | 0 | (0.0%) | 14 | (8.1%) | 50 | (23.9%) | 1 | (0.5%) | | 0 | (0.0%) | | 51 | | (24.4%) | |
| Nausea | 8 | (4.6%) | 3 | (1.7%) | 0 | (0.0%) | 11 | (6.4%) | 7 | (3.3%) | 5 | | (2.4%) | 0 | | (0.0%) | | 12 | | (5.7%) |
| Late period | | | | | | | | | | | | | | | | | | | | |
| Breast pain | 74 | (42.8%) | 3 | (1.7%) | 0 | (0.0%) | 77 | (44.5%) | 79 | (37.8%) | 16 | | (7.7%) | 0 | | (0.0%) | | 95 | | (45.5%) |
| Pigmentation | 43 | (24.9%) | 0 | (0.0%) | 0 | (0.0%) | 43 | (24.9%) | 105 | (50.2%) | 2 | | (1.0%) | 0 | | (0.0%) | | 107 | | (51.2%) |
| Breast swelling | 9 | (5.2%) | 0 | (0.0%) | 0 | (0.0%) | 9 | (5.2%) | 78 | (37.3%) | 6 | | (2.9%) | 0 | | (0.0%) | | 84 | | (40.2%) |
| Dermatitis | 30 | (17.3%) | 5 | (2.9%) | 0 | (0.0%) | 35 | (20.2%) | 19 | (9.1%) | 13 | | (6.2%) | 0 | | (0.0%) | | 32 | | (15.3%) |
| Fibrosis | 16 | (9.2%) | 0 | (0.0%) | 0 | (0.0%) | 16 | (9.2%) | 26 | (12.4%) | 0 | | (0.0%) | 0 | | (0.0%) | | 26 | | (12.4%) |
| Rib change | 9 | (5.2%) | 0 | (0.0%) | 0 | (0.0%) | 9 | (5.2%) | 10 | (4.8%) | 1 | | (0.5%) | 0 | | (0.0%) | | 11 | | (5.3%) |
| Telangiectasia | 5 | (2.9%) | 1 | (0.6%) | 0 | (0.0%) | 6 | (3.5%) | 3 | (1.4%) | 0 | | (0.0%) | 0 | | (0.0%) | | 3 | | (1.4%) |
| Lymphedema | 4 | (2.3%) | 0 | (0.0%) | 0 | (0.0%) | 4 | (2.3%) | 6 | (2.9%) | 0 | | (0.0%) | 0 | | (0.0%) | | 6 | | (2.9%) |
| Pneumonitis | 0 | (0.0%) | 2 | (1.2%) | 0 | (0.0%) | 2 | (1.2%) | 0 | (0.0%) | 5 | | (2.4%) | 3 | | (1.4%) | | 8 | | (3.8%) |
| Fatty necrosis | 4 | (2.3%) | 0 | (0.0%) | 0 | (0.0%) | 4 | (2.3%) | 3 | (1.4%) | 0 | | (0.0%) | 0 | | (0.0%) | | 3 | | (1.4%) |

Abbreviation: APBI, accelerated partial breast irradiation; AWBI, accelerated whole-breast irradiation.

Table S4: Prognostic factors for late toxicity in subgroup analysis (excluding patients who underwent chemotherapy or anti-HER2 therapy)

|  | |  |  | Univariate analysis | | | Multivariate analysis | | |
| --- | --- | --- | --- | --- | --- | --- | --- | --- | --- |
|  |  | No. of events | No. of patients | OR | 95% CI | p-value | OR | 95% CI | p-value |
| Age |  |  |  |  |  | 0.782 |  |  |  |
|  | <60 years | 91 | 116 | 1 | Reference |  |  |  |  |
|  | ≥60 years | 212 | 266 | 1.08 | 0.63-1.84 |  |  |  |  |
| Histology |  |  |  |  |  | 0.103 |  |  |  |
|  | DCIS | 7 | 58 | 1 | Reference |  |  |  |  |
|  | IDC | 69 | 302 | 0.46 | 0.20-1.07 |  |  |  |  |
|  | Other | 3 | 22 | 0.87 | 0.20-3.71 |  |  |  |  |
| Tumor size |  |  |  |  |  | 0.427 |  |  |  |
|  | <1.5 cm | 195 | 242 | 1 | Reference |  |  |  |  |
|  | ≥1.5 cm | 108 | 140 | 0.82 | 0.49-1.35 |  |  |  |  |
| Axillary LN assessment |  |  |  |  |  | 0.842 |  |  |  |
|  | None | 30 | 37 | 1 | Reference |  |  |  |  |
|  | SLNB | 270 | 342 | 0.86 | 0.37-2.07 |  |  |  |  |
|  | ALND | 2 | 3 | 0.47 | 0.04-5.90 |  |  |  |  |
| Radiotherapy technique |  |  |  |  |  | <0.001 |  |  | 0.269 |
|  | APBI | 121 | 173 | 1 | Reference |  | 1 | Reference |  |
|  | AWBI | 182 | 209 | 2.90 | 1.72-4.87 |  | 1.58 | 0.70-3.53 |  |
| Boost radiation |  |  |  |  |  | <0.001 |  |  | 0.071 |
|  | No | 154 | 215 | 1 | Reference |  | 1 | Reference |  |
|  | Yes | 149 | 167 | 3.28 | 1.85-5.81 |  | 2.26 | 0.93-5.47 |  |
| Endocrine therapy |  |  |  |  |  | 0.085 |  |  |  |
|  | No | 39 | 44 | 1 | Reference |  |  |  |  |
|  | Yes | 264 | 338 | 0.46 | 0.17-1.20 |  |  |  |  |

Abbreviations: DCIS, ductal carcinoma in situ; IDC, invasive ductal carcinoma; LN, lymph node; SLNB, sentinel lymph node biopsy; ALND, axillary lymph node dissection; APBI, accelerated partial breast irradiation; AWBI, accelerated whole-breast irradiation.

Table S5 Prognostic factors for grade ≥2 late toxicity in subgroup analysis (excluding patients who underwent chemotherapy or anti-HER2 therapy)

|  | |  |  | Univariate analysis | | | Multivariate analysis | | |
| --- | --- | --- | --- | --- | --- | --- | --- | --- | --- |
|  |  | No. of events | No. of patients | OR | 95% CI | p-value | OR | 95% CI | p-value |
| Age |  |  |  |  |  | 0.976 |  |  |  |
|  | <60 years | 16 | 116 | 1 | Reference |  |  |  |  |
|  | ≥60 years | 37 | 266 | 1.01 | 0.54-1.90 |  |  |  |  |
| Histology |  |  |  |  |  | 0.123 |  |  |  |
|  | DCIS | 12 | 58 | 1 | Reference |  |  |  |  |
|  | IDC | 40 | 302 | 0.59 | 0.29-1.20 |  |  |  |  |
|  | Other | 1 | 22 | 0.18 | 0.02-1.50 |  |  |  |  |
| Tumor size |  |  |  |  |  | 0.860 |  |  |  |
|  | <1.5 cm | 33 | 242 | 1 | Reference |  |  |  |  |
|  | ≥1.5 cm | 20 | 140 | 1.06 | 0.58-1.92 |  |  |  |  |
| Axillary LN assessment |  |  |  |  |  | 0.265 |  |  |  |
|  | None | 8 | 37 | 1 | Reference |  |  |  |  |
|  | SLNB | 44 | 342 | 0.15 | 0.23-1.25 |  |  |  |  |
|  | ALND | 1 | 3 | 0.64 | 0.15-22.63 |  |  |  |  |
| Radiotherapy technique |  |  |  |  |  | <0.001 |  |  | 0.019 |
|  | APBI | 9 | 173 | 1 | Reference |  | 1 | Reference |  |
|  | AWBI | 44 | 209 | 4.86 | 2.30-10.28 |  | 3.55 | 1.24-10.22 |  |
| Boost radiation |  |  |  |  |  | <0.001 |  |  | 0.491 |
|  | No | 16 | 215 | 1 | Reference |  | 1 | Reference |  |
|  | Yes | 37 | 167 | 3.54 | 1.89-6.62 |  | 1.37 | 0.56-3.37 |  |
| Endocrine therapy |  |  |  |  |  | 0.035 |  |  | 0.540 |
|  | No | 11 | 44 | 1 | Reference |  | 1 | Reference |  |
|  | Yes | 42 | 338 | 0.43 | 0.20-0.91 |  | 0.78 | 0.35-1.72 |  |

Abbreviation: DCIS, ductal carcinoma in situ; IDC, invasive ductal carcinoma; LN, lymph node; SLNB, sentinel lymph node biopsy; ALND, axillary lymph node dissection; APBI, accelerated partial breast
